# Gut Microbiota-Derived Resveratrol Metabolites, Dihydroresveratrol and Lunularin, Significantly Contribute to the Biological Activities of Resveratrol

**DOI:** 10.3389/fnut.2022.912591

**Published:** 2022-05-11

**Authors:** Fang Li, Yanhui Han, Xian Wu, Xiaoqiong Cao, Zili Gao, Yue Sun, Minqi Wang, Hang Xiao

**Affiliations:** ^1^Department of Food Science, University of Massachusetts-Amherst, Amherst, MA, United States; ^2^Department of Kinesiology and Health, Miami University, Oxford, OH, United States; ^3^Department of Tea and Food Science, Anhui Agricultural University, Hefei, China

**Keywords:** resveratrol, biotransformation, metabolites, gut microbiota, biological activities

## Abstract

Although resveratrol (RES) is barely detectable in the plasma and tissues upon oral consumption, collective evidence reveals that RES presents various bioactivities *in vivo*, including anti-inflammation and anti-cancer. This paradox necessitates further research on profiling and characterizing the biotransformation of RES, as its metabolites may contribute profound biological effects. After 4-week oral administration, 11 metabolites of RES were identified and quantified in mice by HPLC-MS/MS, including dihydro-resveratrol (DHR), lunularin (LUN), and conjugates (sulfates and glucuronides) of RES, DHR and LUN. Importantly, DHR, LUN, and their conjugates were much more abundantly distributed in tissues, gastrointestinal tract (GIT), and biological fluids compared to RES and its conjugates. Moreover, we established that DHR and LUN were gut bacteria-derived metabolites of RES, as indicated by their depletion in antibiotic-treated mice. Furthermore, the biological activities of RES, DHR, and LUN were determined at physiologically relevant levels. DHR and LUN exhibited stronger anti-inflammatory and anti-cancer effects than RES at the concentrations found in mouse tissues. In summary, our study profiled the tissue distribution of the metabolites of RES after its oral administration in mice and uncovered the important role of gut microbial metabolites of RES in the biological activities of RES *in vivo*.

## Introduction

Resveratrol (*trans*-3, 5, 4′- trihydroxystilbene, RES) is a phytochemical abundant in grapes, wines, peanuts, and mulberries. Voluminous studies have reported the beneficial effects of RES against various chronic diseases, including colitis (1, 2) cardiovascular diseases (3, 4), diabetes (5, 6), and renal diseases (7, 8). However, previous studies demonstrated that only a trace amount of RES could be detected in the plasma and organs upon consumption in both humans and animals, which was presumably due to its extensive metabolism *in vivo* (9–14). Therefore, further studies are warranted to fully profile and characterize the metabolic fate of RES, as its biotransformation may yield metabolites with significant biological activities.

Upon oral consumption, dietary polyphenols are subjected to complex and dynamic biotransformation in the different compartments of the gastrointestinal tract (GIT). In the upper GIT (stomach and small intestine), polyphenols are metabolized by various enzymes, such as cytochrome P450 superfamily enzymes, sulfotransferases, and UDP-glucuronosyltransferases, to form conjugated metabolites (15). Unabsorbed polyphenols and their conjugates reach lower GIT (cecum and colon) and interact with gut microbiota (16). Previous studies mainly focused on the distribution and biological function of RES conjugates in the circulation and peripheral tissues, but not GIT (11, 13, 17, 18), even though, more than 65% of RES-derived metabolites were recovered in GIT (12). Therefore, understanding the dynamic biotransformation of RES and the distribution of its metabolites in different segments of GIT is critical to eliciting its biological effects. Especially, the bioconversion of RES by gut microbiota in lower GIT should not be disregarded (19).

Dihydroresveratrol (DHR), lunularin (LUN) and 3,4′ - dihydroxy-trans-stilbene have been identified as gut microbiota-derived metabolites of RES *via in vitro* fermentation experiments (20). However, important knowledge gaps remain unclarified, such as (1) the relative abundance of these gut microbiota-derived metabolites compared to RES in different tissues; (2) the biological activities of these gut microbiota-derived metabolites compared to RES at physiologically achievable concentrations.

We herein attempt to concretely dissect the biotransformation of RES in GIT and depict the distribution and abundance of its metabolites in biological fluids and peripheral tissues after long-term oral consumption of RES. In addition, this study established the roles of gut microbiota in the biotransformation of RES using both *in vitro* fermentation model and an antibiotic-treated mouse model. Furthermore, based on the concentration of RES, DHR, and LUN found in the kidney and colon, we compared the anti-proliferative, anti-clonogenic, and anti-inflammatory activities of DHR and LUN to that of RES. The current study provided comprehensive insights into the metabolic process of RES occurring in the GIT. Importantly, our findings supported that gut microbiota-derived metabolites, DHR and LUN play important role in the biological activities of RES *in vivo*.

## Materials and Methods

### Materials

Resveratrol (>99% purity) was purchased from Quality Phytochemicals (Edison, NJ, USA). Pinostilbene (PIN; >98% purity) and DHR (>98% purity) were obtained from Yuanye Bio-Technology Co., Ltd (Shanghai, China). LUN (>98% purity) was purchased from Aikon Biopharma LLC (Nanjing, China). Sulfatase (type H-1, from Helix pomatia, containing sulfatase and β-glucuronidase) was obtained from Sigma-Aldrich (St. Louis, MO, USA). Acetonitrile, methanol, acetic acid, and ethyl acetate were purchased from Fisher Scientific (Fairlawn, NJ, USA). All these solvents are HPLC grade. Dimethyl sulfoxide (DMSO) and 3-(4,5-dimethyl2-thizolyl)-2,5-diphenyl-2H-tetrazolium bromide (MTT) were purchased from Sigma-Aldrich (St Louis, MO, USA).

### Animal Experiments

All animal experiments were approved by the Institutional Animal Care and Use Committee of the University of Massachusetts Amherst. Twenty male CD-1 mice (6-week-old) were obtained from Charles River Laboratory (Wilmington, MA, USA). After 1 week of acclimation, 10 mice were randomly chosen to receive a standard AIN93G diet containing 0.05% (w/w) RES (human equivalent dose of 4.6 mg/kg/day), while the other 10 remained on the standard diet. Urine and feces were collected with metabolic cages. All mice were sacrificed with CO_2_ asphyxiation after 4 weeks. The liver, kidney, stomach, small intestine, cecum, colon, and bile were collected and stored at −80°C for further analysis. The small intestine was transversely cut equally into four parts, labeled as SI-1, 2, 3, and 4 referred to as the duodenum, jejunum, proximal ileum, and distal ileum in human. Blood samples were centrifuged at 3,000 g for 15 min at 4°C to collect serum.

In the antibiotic experiment, eight male CD-1 mice (6-week-old) were purchased from Charles River Laboratory (Wilmington, MA, USA) and housed individually. After 1 week of acclimation, all mice received RES enriched diet for 5 days, which contained 0.025% (w/w) RES in the standard AIN93G diet. From day 6, all mice were continuously fed with RES enriched diet but received antibiotic water, which was supplemented with broad-spectrum antibiotics (1.0 g/L ampicillin and 0.5 g/L neomycin) (21). On day 10, all mice were sacrificed with CO_2_ asphyxiation. The urine and fecal samples were collected on day 5 and day 10 with Labsand (Braintree, MA, USA).

### Sample Preparation

Serum, bile, and urine samples were extracted according to previous protocol with minor modification (22). Briefly, samples were vortex-mixed with acidified (2.5% acetic acid) acetonitrile and stood at ice for 20 min to precipitate protein. After centrifugation (14,000 rpm, 10 min, 4°C, Thermo Fisher Scientific), the supernatant was evaporated to dryness under vacuum. Tissue samples were prepared based on previous protocol (13). Briefly, tissues were homogenized by bead ruptor (OMNI, CA, USA) with methanol/water/acetic acid (80:20:2.5) solution, followed by centrifugate at 14,000 rpm for 5 min. Especially for kidney samples, the homogenate was sonicated for 20 min before centrifugation. The residues were extracted one more time and the pooled methanol layers were evaporated to dryness by speed vacuum (SPD111V-120SpeedVAC, Thermo Scientific, MA, USA). The internal standard PIN (5 μmol/L) was spiked to all samples during extraction. All sulfated and glucuronide metabolites were measured by enzymatic hydrolysis of the processed samples with β-glucuronidase and sulfatase as reference described (20).

### Orbitrap Fusion HPLC-MS/MS and HPLC-MS Analysis

The metabolites were eluted with a Zorbax SB-Aq C18 column (Agilent Technologies, Santa Clara, CA, USA) at a flow rate of 0.6 ml/min. Mobile phase A was 5% acetonitrile in water, mobile phase B was 100% acetonitrile. Gradient elution started at 15% solvent B, linear gradient from 15 to 70% solvent B over 18 min, held at 70% B for 3 min, followed by washing and reconditioning the column. The Mass-spectra conditions were optimized at negative electrospray ionization mode, as follows: ion spray voltage 3.5 kv, ion transfer tube temperature 325°C, vaporizer temperature 275°C, sheath gas 15 Arb, aux gas 6 Arb, Orbitrap resolution 120K, and collision energy 30%. Data acquisition and processing were accomplished using Xcalibur V4.1 (Thermo Scientific, MA, USA).

The identified metabolites of RES were quantified by using the Shimadzu Model 2020 HPLC-MS (Shimadzu, Kyoto, Japan). The conditions of chromatography and Mass-Spectra were the same as those of Orbitrap Fusion HPLC-MS/MS. The data was processed with Labsolutions Software (Shimadzu, Kyoto, Japan).

### *In vitro* Fermentation

Pooled fecal samples (3–4 mice) were collected from the cecum and colon of mice that were fed with a standard diet and placed into the anaerobic chamber (A35 anaerobic workstation, Whitley, USA) immediately. Aliquots of fecal samples were suspended in Gifu Anaerobic Broth (GAM, Hemedia, PA, USA). Pooled small intestine digesta were collected from mice fed with RES for 4 weeks and incubated with the fecal suspension under anaerobic conditions for 48 h. Digesta was defined as the complex aqueous suspensions of undigested matters and solubilized nutrients in the GIT lumen (23). Small intestine digesta incubated for 48 h in GAM without fecal microbiota was used as controls. Forty-eight hours later, the fermenta were collected and extracted with ethyl acetate for HPLC-MS analysis (24).

### Cell Viability Assay, Colony Formation Assay, Nitric Oxide Measurement, and TLR-4 Reporter Assay

Human colon adenocarcinoma cell line HT-29 (HTB-38), colorectal carcinoma cell line HCT-116 (CCL-247), renal carcinoma cell line A498 (HTB-44), and renal adenocarcinoma cell line 786-O (CRL-1932) were purchased from American Type Cell Collection (ATCC, Manassa, CA). Mouse macrophage RAW264.7 (TIB-71) was obtained from ATCC. HT-29, HCT-116, A498, and 786-O were subjected to MTT and colony formation assays as described previously to explore the anti-proliferative and anti-clonogenic effects of RES, DHR, and LUN against cancer cell lines (25, 26). Reactive oxygen species production was measured as previously reported with LPS stimulated-RAW264.7 macrophage model (27). Briefly, 10 × 10^4^/well of RAW264.7 cells were incubated in a 96-well back plate for 24 h in RPMI media supplemented with LPS and RES metabolites at stated concentrations. The cells were then washed with PBS and incubated with 100 μl of 10 μM 2′,7′ - dichlorofluorescin diacetate in PBS for 30 min in dark. Subsequently, 100 μl of 0.3 mM of *tert*-butyl hydroperoxide in PBS were added and incubated for 1 h. The oxidized 2′,7′- dichloroflurorescin was measured at the excitation wavelength of 485 nm and the emission wavelength of 528 nm using microplate reader (BioTeck Instrument, Winooski, VT, USA). HEK-Blue™ mTLR-4 cells, in which murine TLR-4, MD2, CD14 co-receptor genes, and inducible secreted embryonic alkaline phosphatase (SEAP) reporter gene were co-transfected into human HEK293 cells, were purchased from InvivoGen (San Diego, CA). The mTLR-4 cells were employed to investigate if RES, DHR, and LUN suppressed inflammation *via* regulating the TLR4-mediated NF-κB signaling pathway. This experiment was conducted as previously described (28). Briefly, 2 × 10^4^/well of mTLR4 cells were suspend in a 96-well plate for 8 h in the HEK-Blue™ detection medium supplied with LPS and testing metabolites at indicated concentrations. The production of secreted SEAP was assessed by reading absorbance at 620 nm. Notably, the concentrations of RES, DHR, and LUN that have been used in the above assays were determined based on the concentrations detected in the kidney and colon of mice fed with RES for 4 weeks.

### Statistical Analysis

Data were tested for normality using the Shapiro-Wilk normality test and statistical significance was determined using GraphPad Prism 8. Data that passed the normality were analyzed using one-way ANOVA with Tukey *post hoc* test for multiple groups with only one variable tested. Two-way ANOVA with Sidak post-test was used for comparison with multiple variables tested in more than two groups. Data passed normality were shown as mean ± standard error (SEM). *P-*value < 0.05 was considered statistically significant.

## Results and Discussion

### Identification of RES Metabolites

**Figure 1:**
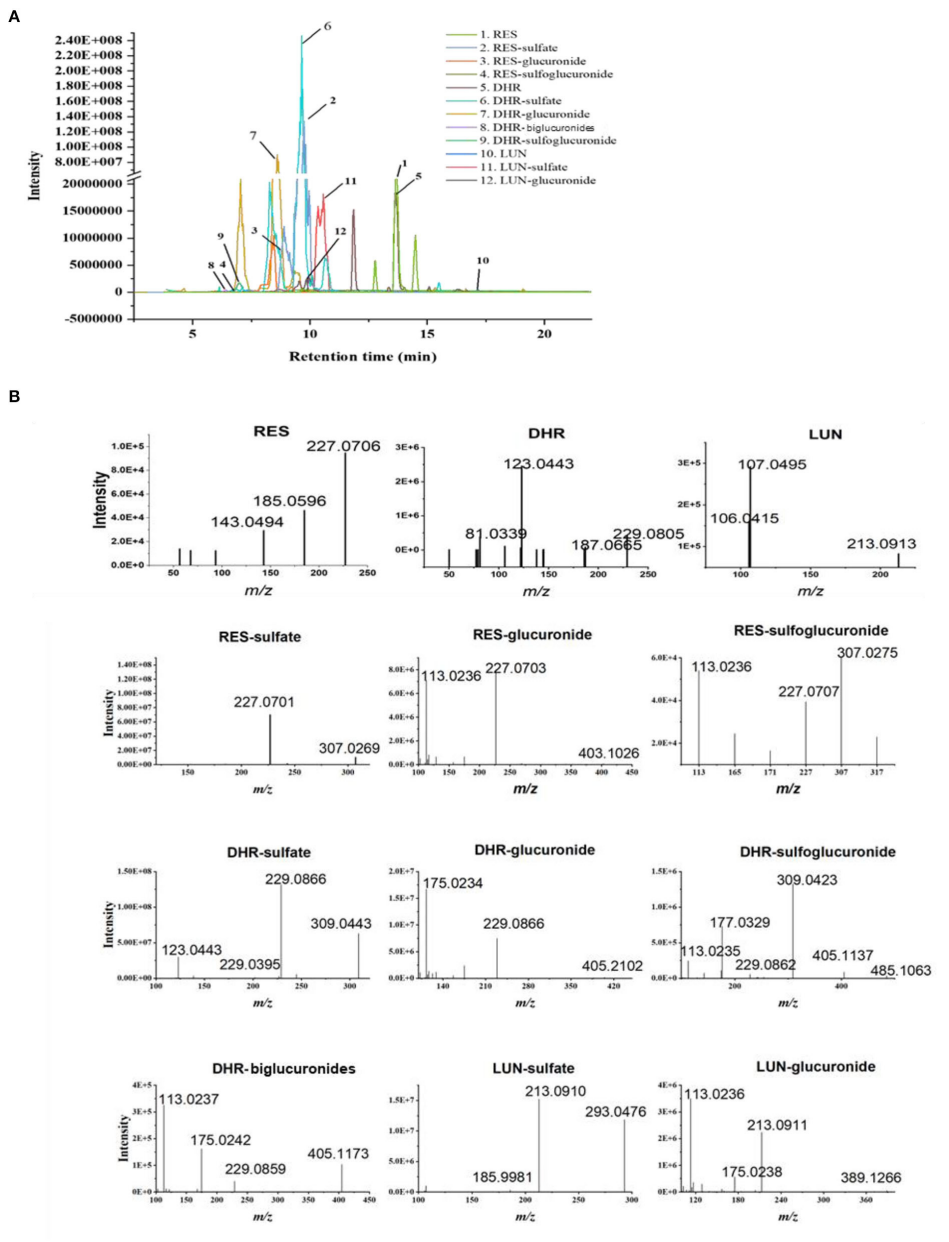
Identification of RES metabolites with HPLC-MS/MS. Chromatograms of 11 RES metabolites identified in the urine and feces after sustained oral consumption of RES with HPLC-MS/MS **(A)**. The MS/MS spectra of RES, DHR, LUN, RES-sulfate, RES-glucuronide, RES-sulfoglucuronide, DHR-sulfate, DHR-glucuronide, DHR-biglucuronides, DHR-sulfoglucuronide, LUN-sulfate and LUN-glucuronide **(B)**.

Resveratrol metabolites in the urine and fecal samples were identified using Orbitrap Fusion Mass Spectrometer. Negative control samples (urine and feces collected from mice fed with a standard diet) were included to eliminate confounding peaks and spectra that were not related to RES-derived metabolites. Eleven metabolites of RES were successfully identified: DHR, LUN, three RES conjugates (RES-sulfate, RES-glucuronide, and RES-sulfoglucuronide), four DHR conjugates (DHR-sulfate, DHR-glucuronide, DHR-biglucuronides, and DHR-sulfate-glucuronide), and two LUN conjugates (LUN-sulfate and LUN-glucuronide). The detailed chromatograms and spectra of RES metabolites were summarized in Figure 1 and Table 1. The retention time (RT) and spectra of RES (RT:13.67 min, [M–H]^−^: 227.0708), DHR (RT:13.42 min, [M–H]^−^: 229.0865), and LUN (RT:17.14 min, [M–H]^−^: 213.0916) were matched with the commercial standards. The rest of the metabolites were identified based on their deprotonated molecular ions and diagnostic product ions (DPIs) within 5 ppm measurement error (Figure 1B and Table 1).

**Table 1:** Metabolites of resveratrol were identified with high-resolution HPLC-MS/MS in the urine.

**#**	**Metabolites**	**Retention time (min)**	**m/z [M–H]^−^**	**MS/MS fragment**
1	RES	13.67	227.071	227.0702, 185.079, 143.0493
2	RES-sulfate	9.75/9.96	307.027	307.0269, 227.0702, 185.0595
3	RES-glucuronide	8.45/6.48	403.103	403.1026, 227.0703, 113.0236, 175.0293
4	RES-sulfoglucuronide	6.86	483.06	307.027, 227.0715, 113.0223
5	DHR	13.42	229.087	229.0837, 123.0442, 81.0339
6	DHR-sulfate	9.65	309.043	309.0424, 229.0395, 123.0443
7	DHR-glucuronide	8.55/8.60	405.119	405.1161, 229.0865, 113.0235
8	DHR-biglucuronides	6.43	581.151	405.1160, 229.0875, 175.0238, 113.0236
9	DHR-sulfoglucuronide	7.01	485.075	485.0757,405.152, 309.0425, 113.0237
10	LUN	17.14	213.092	213.0912, 106.0415, 107.0494
11	LUN-sulfate	10.35/10.56	293.048	293.0476, 213.0910, 79.9567, 107.0494
12	LUN-glucuronide	9.85	389.124	389.1267, 213.0910, 113.0235

Resveratrol-glucuronide, DHR-glucuronide, and LUN-glucuronide possessed deprotonated molecular ions at *m/z* 403.1029, *m/z* 405.1186, and *m/z* 389.1236, respectively. They were 176 Da more than their corresponding parent moieties. In their MS spectra, the DPIs at *m/z* 227.0703, *m/z* 229.0395, *m/z* 213.0912, and *m/z* 175.0293 were detected. Therefore, they were tentatively identified as glucuronidated metabolites. DHR-biglucuronides generated an [M–H]^−^ ion at *m/z* 581.1506. The MS characteristic DPIs at *m/z* 405.1160 and *m/z* 175.0238 revealed that the biglucuronidation most likely occurred to DHR.

Resveratrol-sulfate, DHR-sulfate, and LUN-sulfate showed [M–H]^−^ ions at *m/z* 307.0269, *m/z* 309.0433, and *m/z* 293.0484, respectively. They were 80 Da greater than their parent moieties, which indicated the existence of sulfate moiety. In MS spectra, they yielded DPIs at *m/z* 227.0703, *m/z* 229.0395, and *m/z* 213.0912. Thus, they were tentatively identified as isomeric sulfated metabolites.

Our results suggested that RES were transformed to DHR and LUN, which were consistent with previous findings (10, 11, 29). Subsequently, RES, DHR, and LUN underwent sulfation and glucuronidation to produce their corresponding conjugates (30).

### Distribution of RES and Its Metabolites in Tissues and Biological Fluids

**Figure 2:**
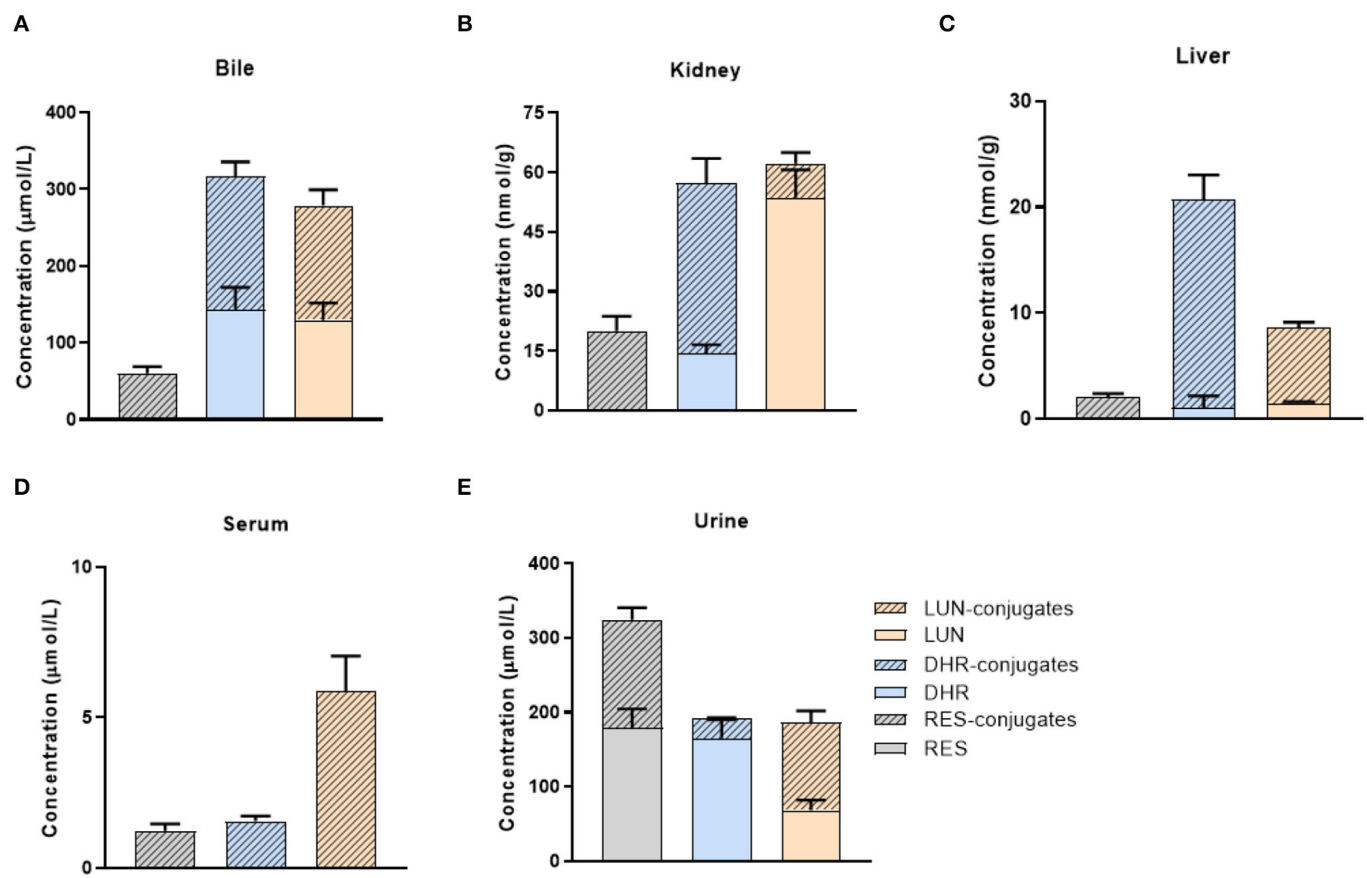
Tissue distribution of RES metabolites. Concentrations of RES, DHR, LUN, and their conjugates in the bile **(A)**, kidney **(B)**, liver **(C)**, serum **(D)**, and urine **(E)**.

We quantified the concentration of RES and its metabolites in the liver, kidney, and biological fluids including urine, serum, and bile in mice. Due to the paucity of available standards for sulfate and glucuronide conjugates, they were semi-quantified by enzymatic hydrolysis (20). RES was not detectable in the liver, kidney, serum, or bile (Figures 2A–D), which indicated that RES underwent extensive metabolism after oral consumption and further emphasized the bioactive potential of its metabolites. Moreover, DHR, LUN, and their conjugates were much more abundant than RES-conjugates (Figures 2A–D). As shown in Figure 2, DHR + DHR-conjugates and LUN + LUN-conjugates were 5.3- and 4.6- folds higher in the bile, 1.2- and 4.8- folds higher in the serum, 10.3- and 3.4- folds higher in the liver, and 2.9- and 3.1-folds higher in the kidney than RES + RES-conjugates, respectively. The above results suggested that besides RES-sulfate, RES-glucuronide, and RES-sulfoglucuronide that were reported previously, DHR, LUN and their conjugates were more abundant metabolites after oral consumption of RES. Higher amounts of DHR + DHR-conjugates than RES + RES-conjugates were reported before (31), while our results for the first time demonstrated the high abundance of LUN and their conjugates in mouse tissues. Considering the absence of RES and the high abundance of DHR, LUN, and their conjugates in tissues, it is reasonable to speculate that these metabolites might play critical roles in biological functions.

Furthermore, high concentrations of RES (179.1 μmol/L) and its conjugates (145.3 μmol/L) were detected in urine (Figure 2E). It suggested that a large amount of RES+RES-conjugates was excreted through urine compared with DHR, LUN and their conjugates, which further supported the results shown in Figures 2A–D. Importantly, the relatively high concentrations of DHR and LUN in the bile should be noted which may be attributed to the reabsorption through the enterohepatic circulation (Figure 2A) (32).

### Distribution of RES and Its Metabolites in GIT

Previous studies mainly focused on the distribution of RES metabolites in peripheral tissues but not in GIT, especially the colon. The extensive metabolism of RES resulted in the accumulation of RES metabolites in the GIT *via* efflux pump and bile secretion, where they might be subjected to substantial biotransformation by gut microbiota. For a better understanding of the dynamic metabolic fate of RES after oral consumption, we quantified the abundance of RES metabolites in both the digesta (inner content) and mucosa of different parts of GIT (stomach, small intestine, cecum and colon).

**Figure 3:**
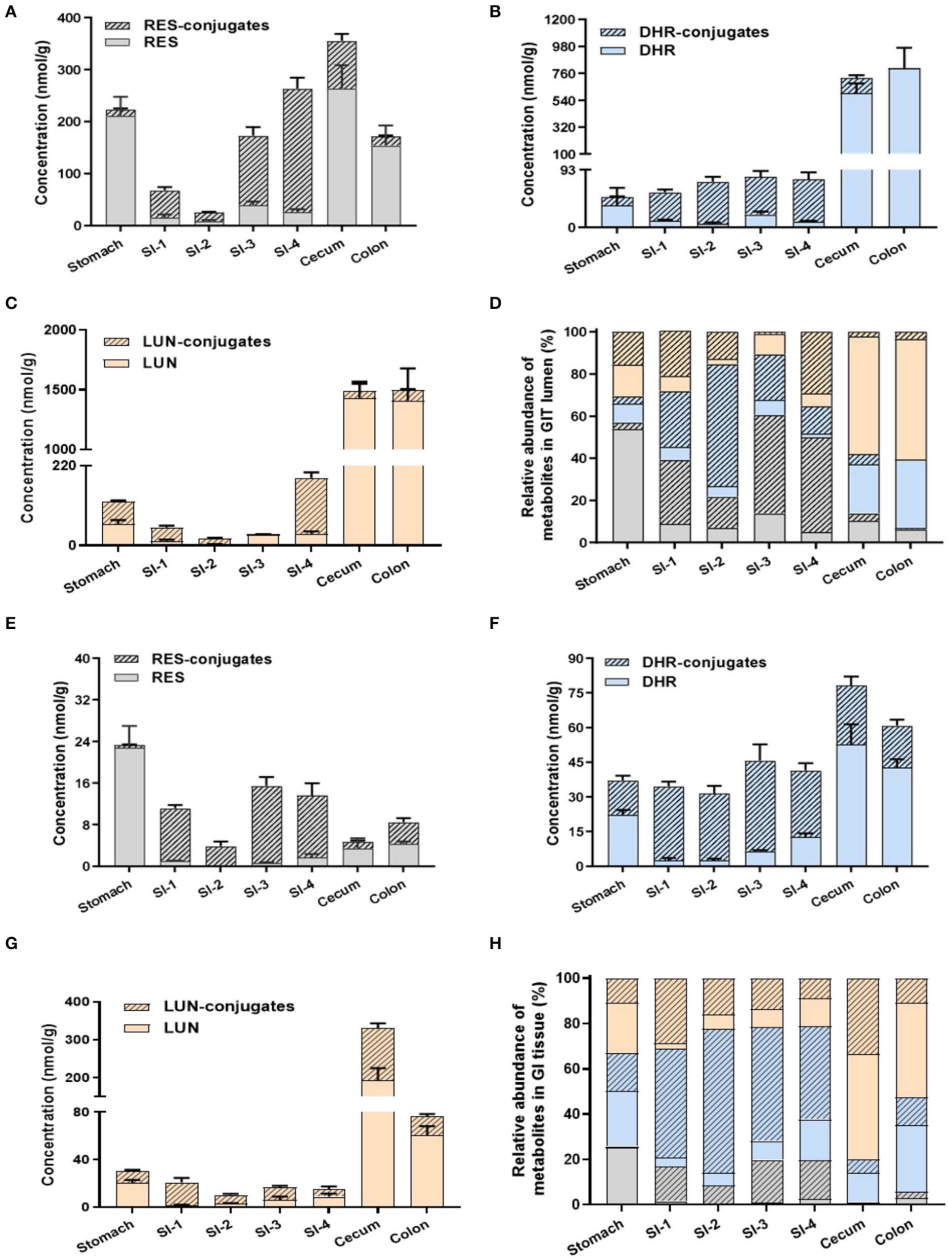
Levels of RES metabolites in gastrointestinal digesta and tissues. Concentrations of RES, DHR, LUN, and their corresponding conjugates in digesta **(A–C)**. Relative abundance of RES metabolites in digesta **(D)**. The concentration of RES, DHR, LUN, and their corresponding conjugates in GIT tissues **(E–G)**. Relative abundance of RES metabolites in GIT tissues **(H)**.

A considerable amount of RES was detected in the stomach digesta, as well as relatively lower levels of RES-conjugates, DHR, DHR-conjugates, LUN, and LUN-conjugates (Figures 3A,D). Conjugates in stomach digesta could attribute to the metabolizing ability of gastric tissue (33). The presence of DHR and its conjugates in the stomach digesta has been reported before by Azorin-Ortuno et al. (12), which was tentatively explained by the presence of microbial groups in the stomach. This may also explain the appearance of LUN and its conjugates in the stomach lumen. Besides, another pivotal reason for the presence of DHR, LUN and their conjugates in the stomach is coprophagia, by which mice obtained considerable amount of DHR and LUN from feces (34).

Resveratrol, DHR, and LUN dominated (78.06%) in the stomach digesta (Figure 3D). However, once the digesta entered the SI, the concentrations of RES, DHR, and LUN showed a dramatic decline, meanwhile, their conjugates significantly increased to 80.81% of total RES metabolites (average of SI-1 to SI-4; Figure 3D). The substantial amount of conjugates in the SI digesta could be attributed to phase II enzymes that are rich in SI enterocytes and continuous influx of biliary conjugates through the enterohepatic circulation (12).

Intriguingly, when digesta moved from the last segment of SI (SI-4) to the cecum the relative abundance of RES-conjugates, DHR-conjugates, and LUN-conjugates decreased from 44.95, 12.98% and 29.15 to 3.56%, 4.83 and 2.17%, respectively (Figures 3A–D). When it further moved down to the colon, the relatively abundance of RES-conjugates, DHR-conjugates, and LUN-conjugates accounted for 0.73, 0.01, and 3.48% of the total metabolites, respectively (Figures 3A–D). Meanwhile, the relative abundance of their corresponding parent compound, RES, DHR and LUN, were dramatically increased to 6.22, 32.45, 57.10% in the colon. The cecum and colon host high diversity and density of gut microbiota, which may contribute to the process of deconjugate. A previous study reported that oral administration of RES-sulfate resulted in a detectable level of RES in the plasma, supporting the deconjugate process (35).

The distribution of RES and its metabolites in the GIT mucosa had a similar pattern with GIT digesta, as shown in Figures 3E–H. It is noteworthy that the overall concentration of RES and its metabolites were considerably lower in GIT mucosa compared to digesta. Interestingly, DHR and LUN, rather than RES, were the major metabolites detected in colonic mucosa, indicating DHR and LUN may significantly contribute to the health effects of RES in the colonic diseases.

### The Role of Gut Microbiota in the Biotransformation of RES

**Figure 4:**
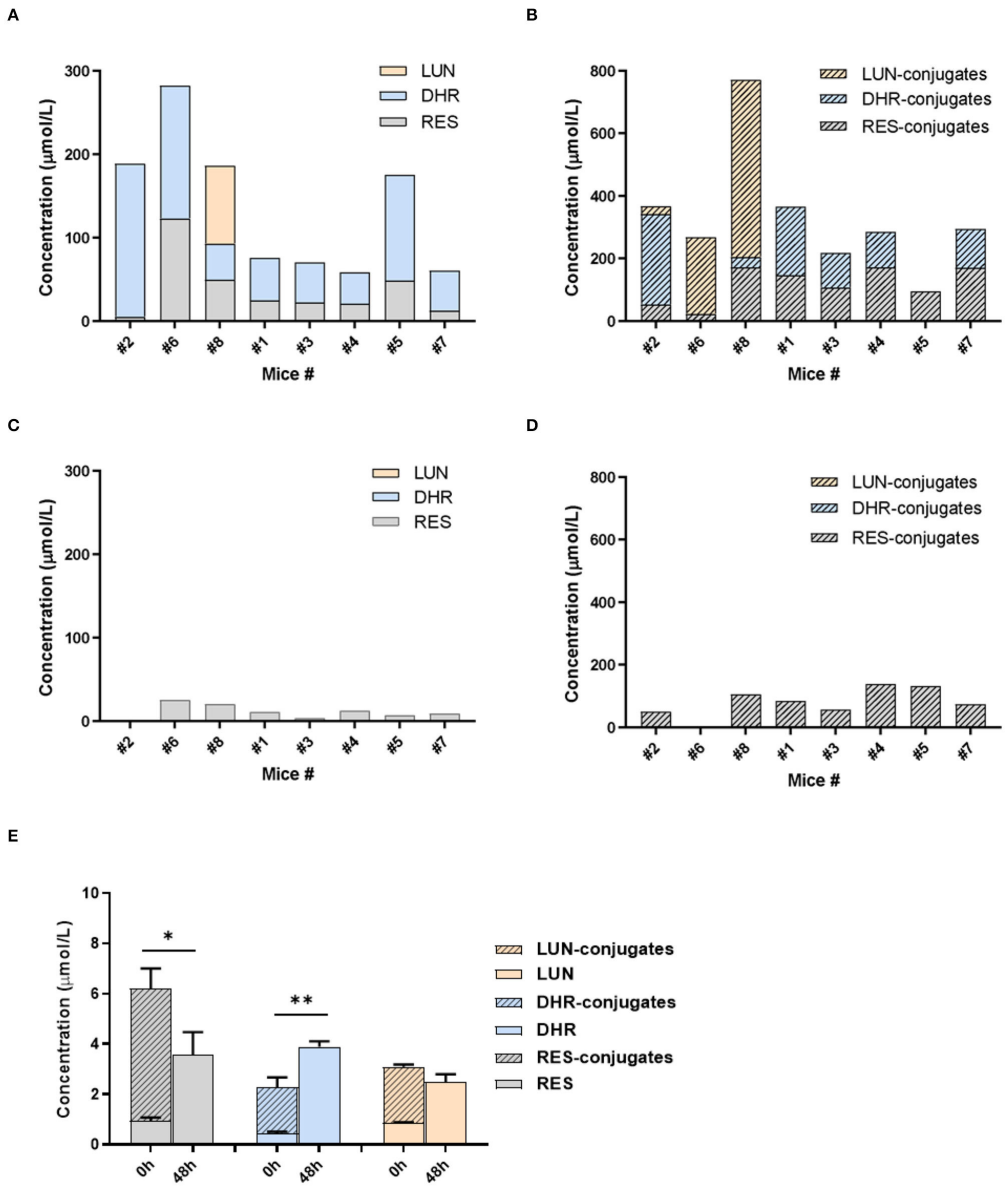
Gut microbiota-mediated RES biotransformation. The concentrations of RES, DHR, LUN, and their conjugates in the urine before antibiotic treatment **(A,B)**. The concentrations of RES, DHR, LUN, and their conjugates in the urine after antibiotic treatment **(C,D)**. The concentration of RES, DHR, LUN, and their conjugates in SI digesta before and after anaerobic fermentation with mouse fecal bacteria **(E)**. Three independent experiments were performed **(E)**. Significant differences are indicated: *P < 0.05, **P < 0.01 **(E)**. Mice were numbered as number (#) 1, #2, #3 …to #8 in our experiment, which was described in the text of “The Role of Gut Microbiota in the Biotransformation of RES”.

Given the distinct metabolic patterns of RES in small intestine vs. large intestine, we hypothesized that gut microbiota plays an important role in the deconjugation of phase II metabolites and production of DHR and LUN in the cecum and colon. To appreciate the interindividual differences in the composition/function of gut microbiota, mice were individually caged. The metabolites of RES in urine were measured before and after antibiotic treatment in the mice. As shown in Figures 4A,B, all eight mice could transform RES to DHR with variation, while only three mice could produce LUN before antibiotic treatment. LUN was a trace metabolite in mice #2, which only account for <5% of the total metabolites. While DHR and its conjugates account for 84.75% of the metabolites in #2 mice (Figures 4A,B). In mice #6 and #8, LUN and its conjugates accounted for 44.48 and 68.82% of the metabolites in the urine, respectively. These results could attribute to the interindividual differences in gut microbiota. In the other five mice, RES-conjugates, DHR, and DHR-conjugates were major metabolites with variation in abundance (Figures 4A,B).

Our previous study showed that antibiotic treatment could decrease the abundance of mouse gut microbiota by more than 500 folds (36), therefore, diminish the metabolic function of gut microbiota. Herein, we showed that DHR, LUN, and their conjugates completely disappeared after 5 days of antibiotic treatment (compare Figures 4A–D). These results demonstrated that DHR and LUN were gut microbial metabolites of RES. These findings provided fundamental information on the metabolic pathway of RES after oral consumption in human. The pronounced interindividual differences in gut microbiota should be taken into account during the investigation of health-related effects of RES and other dietary compounds in the future. Moreover, it is important to understand the alteration of gut microbiota composition and its implications in the biotransformation of dietary compounds in the context of human health and diseases.

**Figure 5:**
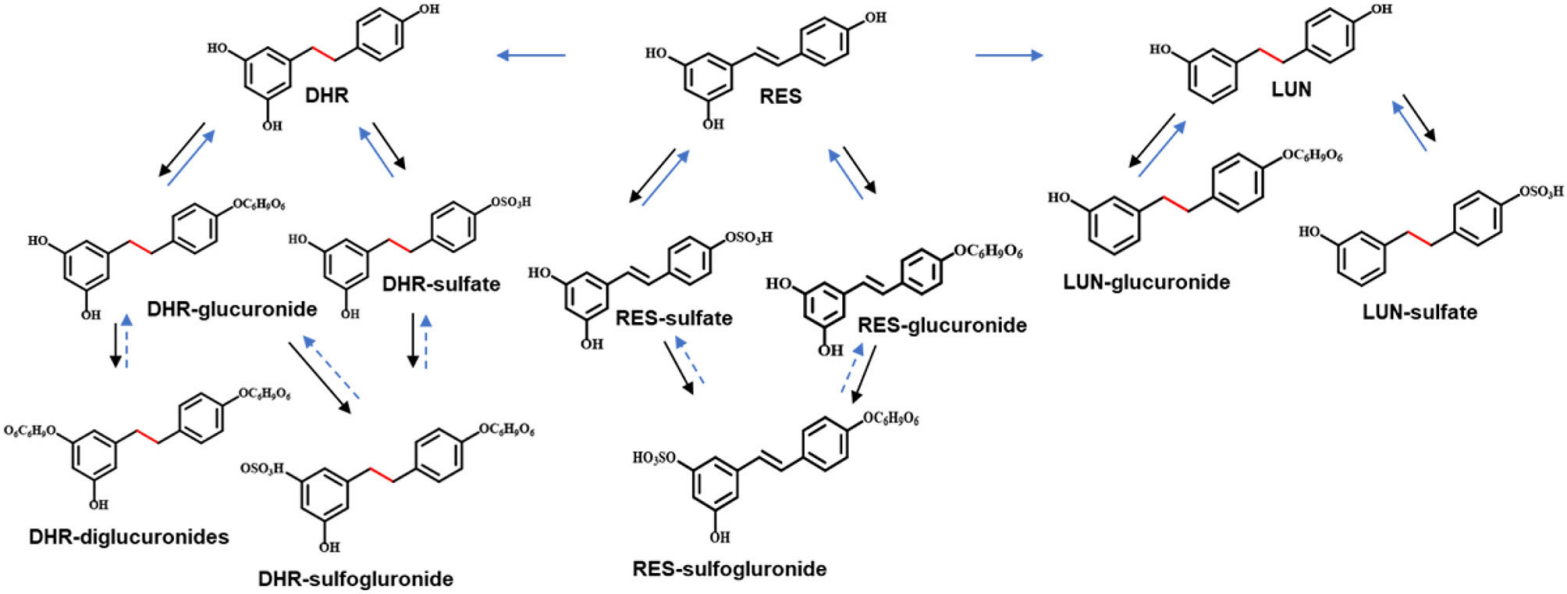
Proposed metabolic pathway of RES. Solid arrows in black indicated metabolism conducted by digestive enzymes. Solid arrows in blue indicated the involvement of gut microbiota. Arrows in dashed lines represented speculated metabolic paths.

To establish the deconjugation role of gut microbiota, we incubated SI digesta collected from RES-fed mice, with gut bacteria obtained from mice fed with a regular diet to determine the levels of free- and conjugated-metabolites before and after the fermentation. After 48 h of anaerobic incubation, the conjugates RES-M, DHR-M and LUN-M completely disappeared and the abundance of RES, DHR and LUN increased (Figure 4E), which established the indispensable role of gut microbiota in the deconjugate reactions of RES-, DHR-, and LUN-conjugates. Importantly, after fermentation the concentration of DHR significantly enhanced compared to the sum of DHR and DHR-conjugates before fermentation (*P* < 0.01, Figure 4E). Meanwhile, the concentration of RES after fermentation was dramatically decreased compared to the total of RES and RES-conjugates before fermentation (P < 0.05, Figure 4E). Above observations indicated that RES could be transformed to DHR by gut microbiota, which was consistent with previous studies and our results in Figures 4A–D (20). The concentration of LUN at 48 h was comparable to the levels of LUN + LUN conjugates before fermentation (Figure 4E), suggesting RES was not converted to LUN in our experimental conditions. Multiple factors, including limited microbial strains could produce LUN (Figures 4A,B), LUN-producible strains were not culturable *in vitro* and LUN might be further transformed to other metabolites that have not been identified, could explain the lack production of LUN (20). Based on the above results, the proposed metabolic fate of resveratrol was summarized in Figure 5.

### DHR and LUN Showed Stronger Anti-cancer and Anti-inflammatory Effects Than RES at Physiological Concentrations

Based on the readout in Figures 2, 3, a limited amount of RES could be detected across the tissues and biological fluids, which encourages the further characterization of the biological activities of RES metabolites. Previous studies revealed that the phase II metabolites of RES such as RES-3-*O*-sulfate and RES-3-*O*-gucuronide only exhibited moderate bioactivities (37, 38). Therefore, we focused on illustrating the bioactivities of two gut microbiota-derived metabolites, DHR and LUN, in this study. RES exhibited protective effects in colitis and renal diseases (2, 8). Meantime, considerable amount of DHR and LUN were detected in the kidney and colon (Figures 2, 3). Thus, the chemopreventive effects of DHR and LUN were examined in renal and colonic cancer cell lines. To establish the protective effects of RES and its metabolites in a physiologically relevant manner, we deliberately used the concentrations found in these tissues to determine their bioactivities.

**Figure 6:**
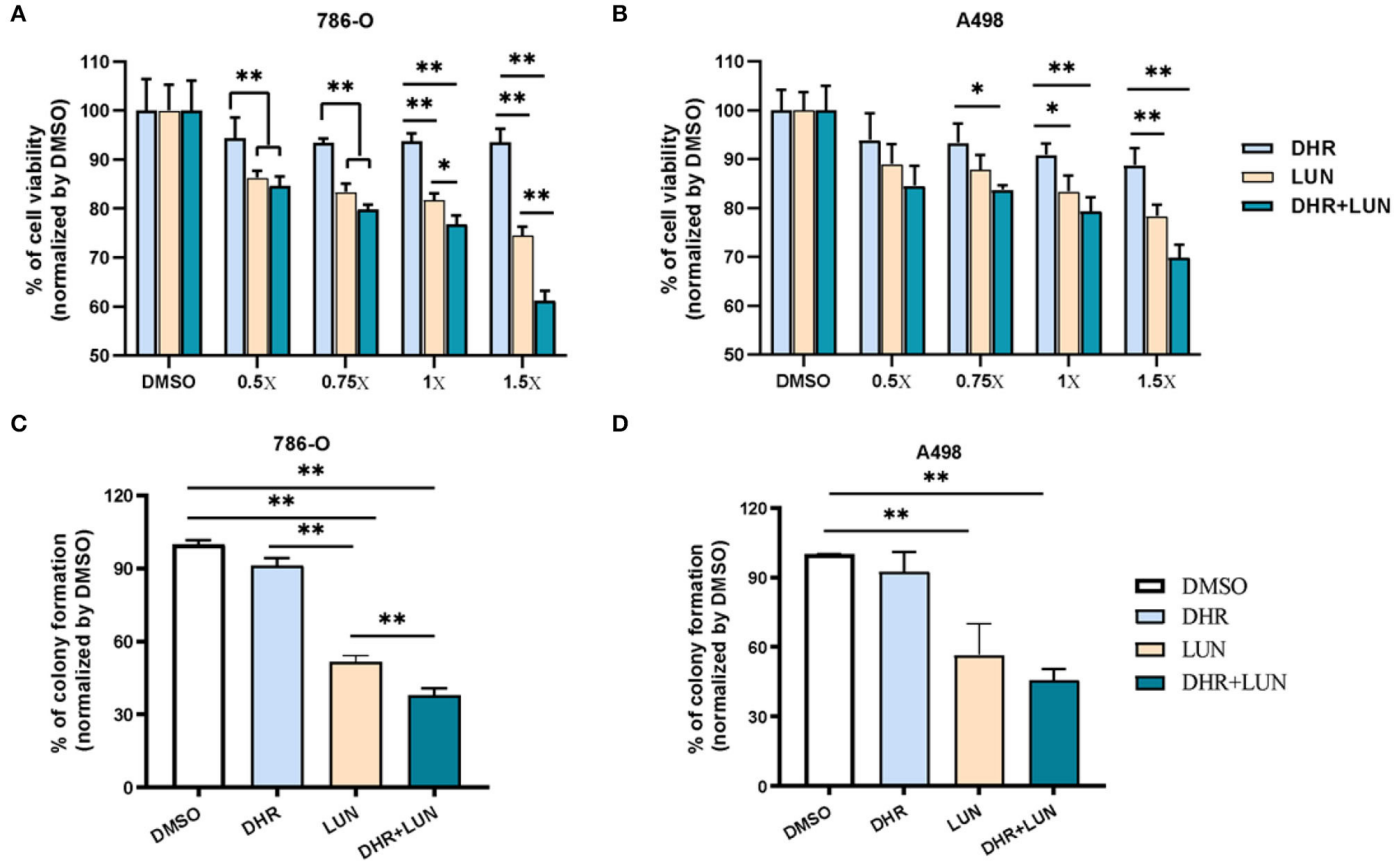
Anti-proliferative and anti-clonogenic effects of DHR and LUN in renal carcinoma cell lines at tissue-relevant concentrations. Anti-proliferative effects of DHR and LUN in 786-O **(A)** and A498 **(B)** renal carcinoma cells. Anti-clonogenic effects of DHR and LUN in 786-O **(C)** and A498 **(D)** cells. Three independent experiments were conducted. Data presented as mean ± SEM. Significant differences are indicated: *P < 0.05, **P < 0.01.

Since RES was not detectable in the kidney, the renal protective effects of RES inevitably pointed to its metabolites. A498, a “classical” human renal carcinoma cell line, is widely used as a model of clear cell renal cell carcinoma (ccRCC) (39). 786-O, with the phenotype of ccRCC, is the primary cell line that is most commonly used in renal carcinoma-focused research (38). These two cell lines were adopted to evaluate the anti-proliferative and anti-clonogenic effects of DHR and LUN at renal relevant concentrations as indicated in Figure 2B. Four levels of DHR, LUN, and DHR + LUN were used at 0.5, 0.75, 1, and 1.5×. Concentration at 1× was equivalent to the concentrations (DHR: 14.3 nmol/g; LUN: 53.6 nmol/g) found in kidney tissues (Figure 2B). Concentrations at 0.5, 0.75, and 1.5× were half, three quarters and one and half times of the concentrations of RES metabolites at 1×, respectively. As shown in Figures 6A,B, LUN, but not DHR, inhibited the proliferation of both 786-O and A498 cells in a dose-dependent manner. LUN showed stronger inhibitory effects than DHR in both 786-O and A498 cells at tested concentrations above 0.75× (*P* < 0.05). LUN caused 15.6, 16.5, 18.2 and 25.4% of inhibitions on 786-O cells at 0.5, 0.75, 1 and 1.5×, respectively (Figure 6A). The combination of DHR and LUN produced stronger inhibitory effects at a concentration of 1×, treatment of DHR+LUN caused 23.2% (*P* < 0.05) death of 786-O cells (Figure 6A). A498 and 786-O cells were also subjected to colony formation assay at the 1× concentration. The colonies were scanned and counted as shown in Figures 6C,D (Supplementary Figure S1). LUN, but not DHR, significantly inhibited the clonogenic formation of A498 and 786-O cells by 43.38 and 48.44%, respectively (*P* < 0.01). A combination of DHR and LUN exhibited stronger inhibitory effects than LUN alone in 786-O (*P* < 0.01, Figure 6C). As shown in Figures 6C,D, DHR + LUN suppressed the colony formation by 54.15 and 62.03% in A498 and 786-O cells, respectively. These results suggested that the renal protective effects of RES were attributed to its gut microbiota-derived metabolites (DHR and LUN) in the kidney.

**Figure 7:**
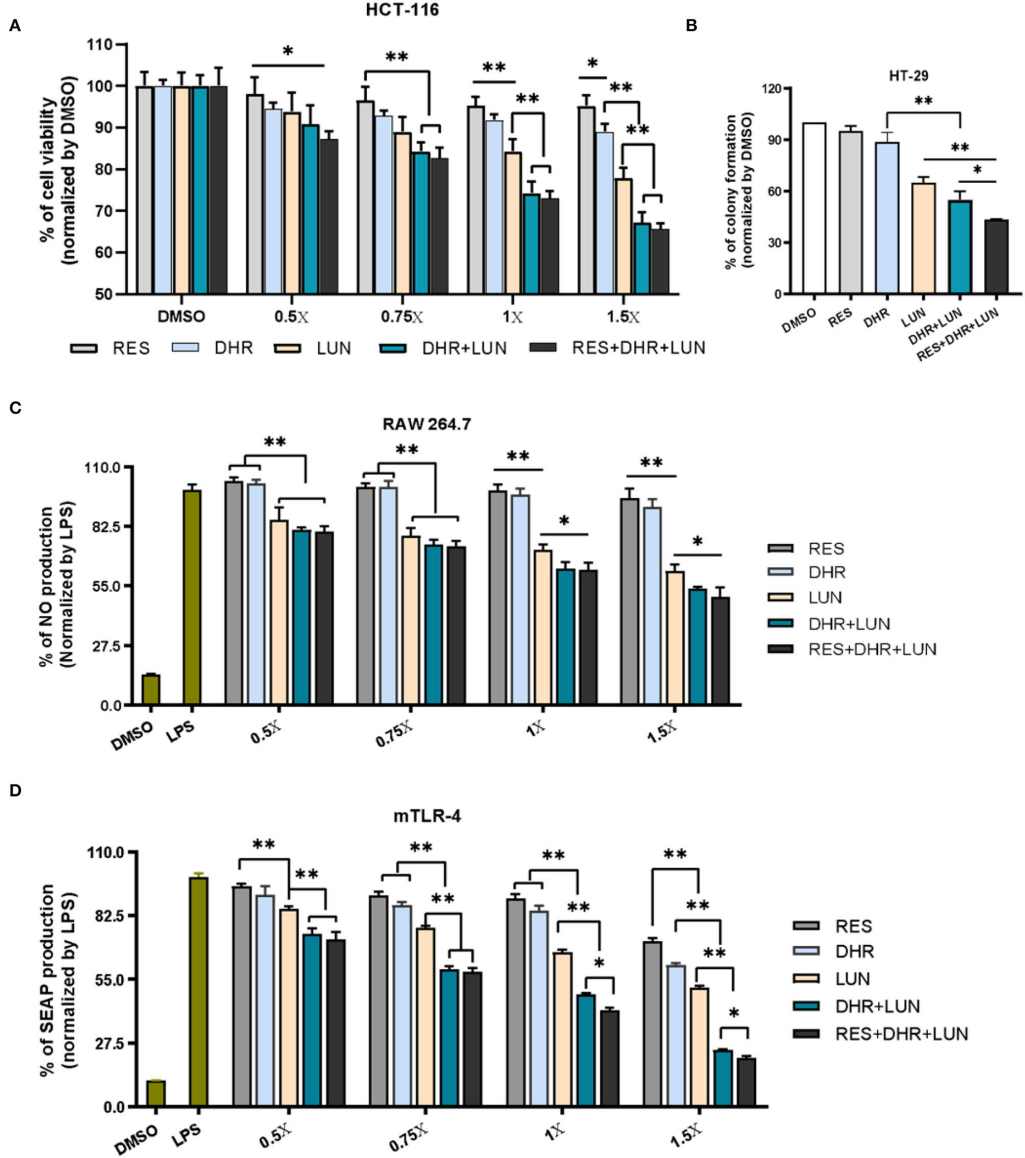
Anti-proliferative, anti-clonogenic, and anti-inflammatory effects of RES, DHR, and LUN at the colonic tissue levels. Anti-proliferative effects of RES, DHR, and LUN on HCT-116 cancer cell line **(A)**. Anti-clonogenic effects of RES, DHR, and LUN on HT-29 cancer cell line **(B)**. The inhibition of RES, DHR, and LUN on NO production in LPS-stimulated RAW264.7 macrophages **(C)**. Inhibitory effects of DHR, LUN, and their combination on LPS-induced SEAP production in HEK™ mTLR-4 cells **(D)**. Three independent experiments were conducted. Data presented as mean ± SEM. Significant differences are indicated: *P < 0.05, **P < 0.01.

To establish anti-colonic cancer potential of DHR and LUN, we determined their anti-proliferative and anti-clonogenic effects on two widely used human colon cancer cell lines (HCT-116 and HT-29) at the concentrations found in the mouse colonic tissues (Figure 3). Treatment of 1× stood for concentrations measured in the colonic tissue, that was, 4.3 nmol/g of RES, 42.8 nmol/g of DHR, and 60.5 nmol/g of LUN. At the tested concentrations, RES did not show any biological effects in all cell types (Figure 7). DHR showed a tendency to suppress the proliferation of HCT-116 cells but did not achieve statistical significance. LUN profoundly inhibited the proliferation of HCT-116 cells at concentrations higher than 1× compared to RES (*P* < 0.01). Markedly, DHR+LUN together showed stronger inhibitory effects than DHR and LUN alone at 1.0×. Compared to 8.2 and 12.7% of inhibition induced by DHR and LUN, DHR+LUN inhibited cell growth by 24.8% (*P* < 0.01) at a concentration of 1.0× (Figure 7A). The combination of all three compounds (RES + DHR + LUN) exerted the strongest inhibition, however, it was marginally greater than that produced by a combination of two metabolites (DHR + LUN). This finding further demonstrated the meager contribution of RES itself to its *in vivo* protective effects against colon cancer. It is noteworthy that at the concentration of 1.5×, DHR + LUN+RES showed no significant inhibition of the growth of normal human colon CCD-18Co cells (Supplementary Figure S2A). This result indicated that cytotoxic effects of RES, DHR, and LUN were cancer cell specific.

HT-29 cells were subjected to different treatments at 1×. After 12 days of incubation, the colonies were photographed and quantified as shown in Figure 7B and Supplementary Figure S2B. The numbers of colonies formed followed the order of RES ≈ DHR > LUN > DHR + LUN > RES + DHR + LUN. RES and DHR alone at tested concentration did not show significant effects on colony formation. Compared to DHR alone, treatments of LUN, DHR + LUN, and RES + DHR + LUN restricted the clonogenic survival of HT-29 cells by 35.2, 45.0, and 56.7%, respectively (*P* < 0.05; Figure 7B). These results indicated that RES metabolites might play more vital roles in inhibiting colon cancer cells than RES itself in the colonic tissue after oral consumption of RES.

Besides the anti-proliferative and anti-clonogenic effects, RES metabolites also exerted a stronger anti-inflammatory ability than RES at the colonic concentrations (Figure 7C). LUN showed a dose-dependent inhibition on LPS-induced NO production (an important inflammatory mediator) by 14.2, 21.6, 28.3, and 38.2% at 0.5, 0.75, 1, and 1.5×, respectively (*P* < 0.01). Overall, DHR did not produce a significant anti-inflammatory effect, which was consistent with the previous study (36). The combined treatment of DHR and LUN caused a significant decrease in the production of NO compared with LUN alone (*P* < 0.05) at concentrations above 1×. The combination of all three compounds (RES, DHR, and LUN) did not produce stronger inhibitory effects in comparison with the combination of DHR and LUN (Figure 6C), suggesting DHR and LUN rather than RES were more important in anti-inflammatory activities in the colon.

To gain further understanding of the anti-inflammatory signaling pathway, we employed mTLR-4 cells to examine if DHR and LUN suppressed inflammation *via* regulating TLR-4 mediated NF-κB pathway. Stimulation of mTLR-4 cells with a bacterial toxin LPS activated NF-κB and activator protein 1 (AP-1), which induces the production of SEAP. As shown in Figure 7D, a single treatment of LUN at all tested concentrations caused a significant dose-dependent inhibition of SEAP production compared to RES alone (*P* < 0.01). Furthermore, cotreatments with serial concentrations of DHR + LUN resulted in suppression of SEAP production by 25.4, 40.6, 51.5, and 75.6% at 0.5, 0.75, 1, and 1.5×, respectively. The involvement of RES strengthened the inhibitory effects of DHR + LUN on SEAP expression but was not statistically significant at lower concentrations (0.5 and 0.75 ×; Figure 7D). Further analyses are required to clarify the specific molecular mechanisms of the anti-inflammatory effects of DHR and LUN. It should be noted that RES exhibited stronger inhibitory effects on cancer cell lines (37) and NO production than DHR and LUN at the same concentration. However, this dose range of RES is not achievable in our *in vivo* feeding study (Supplementary Figure S2C).

Overall, our results demonstrated that LUN exhibited stronger biological activities compared to RES at tissue-relevant levels. DHR alone showed moderate bioactivities in almost all tested cell models. While the combination of DHR and LUN often displayed stronger beneficial effects compared with LUN and DHR alone, suggesting potential synergistic effects that are worth to be investigated in the future. By using physiological relevant concentrations, our study strongly supported that DHR and LUN contributed a great portion of the beneficial effects of RES in renal and colonic diseases. The biological effects of DHR and LUN in renal and colonic diseases need to be validated *in vivo* in the future, which may provide preventative or therapeutic strategies for patients unresponsive to RES due to the lack of proper gut microbial strains.

## Conclusion

The present study systemically elucidated the dynamic biotransformation of RES by focusing on its metabolic fate in the GIT. Eleven metabolites of RES have been successfully identified. The conjugates of RES, DHR, and LUN were dominantly distributed in the SI and largely deconjugated back to their parent compounds in the lower GIT by gut microbiota. Moreover, our antibiotic-treated mouse experiment concluded that DHR and LUN were produced by gut microbiota. Importantly, DHR, LUN, and their combination exerted stronger anti-proliferative and anti-inflammatory effects in the renal and colonic cell lines, at concentrations achievable in these tissues, suggesting that DHR and LUN may significantly contribute to the chemopreventive properties elicited by RES in the kidney and colon. Overall, our findings provided a solid scientific basis for understanding the health effects of RES from the perspective of biotransformation and are of great value for future research on RES in the prevention and treatment of renal and colonic diseases in humans.

## Data Availability Statement

The original contributions presented in the study are included in the article/Supplementary Material, further inquiries can be directed to the corresponding author.

## Ethics Statement

The animal study was reviewed and approved by University of Massachusetts Amherst.

## Author Contributions

FL: data collection, methodology, and writing—original draft preparation. YH: data collection and visualization. XW: investigation. XC: visualization and investigation. ZG and YS: partial data collection. MW: writing—reviewing. HX: conceptualization, validation, and supervision. All authors contributed to the article and approved the submitted version.

## Funding

All sources of funding received for the research are submitted. This work was partially supported by the National Institutes of Health (R01AT010229) and the United States Department of Agriculture (NIFA grants #2019-67017-29249 and 2020-67017-30835).

## Conflict of Interest

The authors declare that the research was conducted in the absence of any commercial or financial relationships that could be construed as a potential conflict of interest.

## Publisher's Note

All claims expressed in this article are solely those of the authors and do not necessarily represent those of their affiliated organizations, or those of the publisher, the editors and the reviewers. Any product that may be evaluated in this article, or claim that may be made by its manufacturer, is not guaranteed or endorsed by the publisher.

## Supplementary Material

Supplementary Figure 1 Representative images of 786-O **(A)** colonies and A498 **(B)** colonies after various treatments.

Supplementary Figure 2 Anti-proliferative effects of DHR and LUN in CCD-18Co cell line with concentrations equivalent to 1.5 times that measured in colonic tissues **(A)**. Representative images of HT29 **(B)** colonies. At the indicated concentrations, RES showed stronger inhibitory effects on NO production compared to DHR and LUN **(C)**. Three independent experiments were conducted. Data presented as mean ± SEM. Significant differences are indicated: *, *P* < 0.05.

## Abbreviations

ccRCC: clear cell renal cell carcinoma
DHR: dihydro-resveratrol
LPS: lipopolysaccharides
LUN: lunularin
NO: nitric oxide
RES: resveratrol
SEAP: secreted embryonic alkaline phosphatase.
