# Supplementary material for: Gut Microbiota-Derived Resveratrol Metabolites, Dihydroresveratrol and Lunularin, Significantly Contribute to the Biological Activities of Resveratrol
Source: Front Nutr. 2022 May 11;9:912591. doi: 10.3389/fnut.2022.912591 (PMC9131081; doi:10.3389/fnut.2022.912591)
Supplement: Supplementary Figure 1 — Representative images of 786-O (A) colonies and A498 (B) colonies after various treatments. [file Presentation_1.PPTX]

## Slide 1
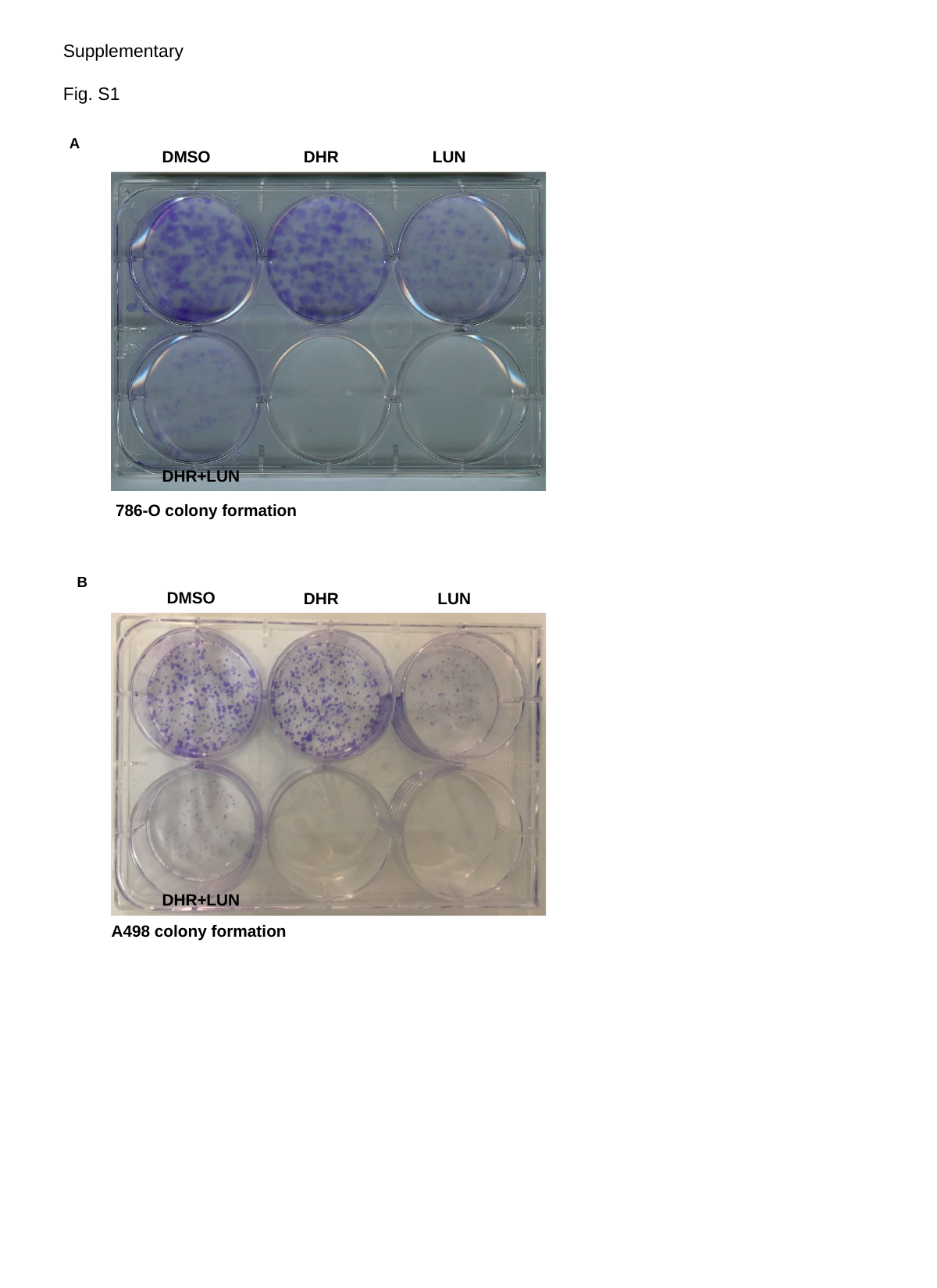

Supplementary
Fig. S1
A
LUN
DMSO
DHR
DHR+LUN
786-O colony formation
B
DMSO
DHR
LUN
DHR+LUN
A498 colony formation

## Slide 2
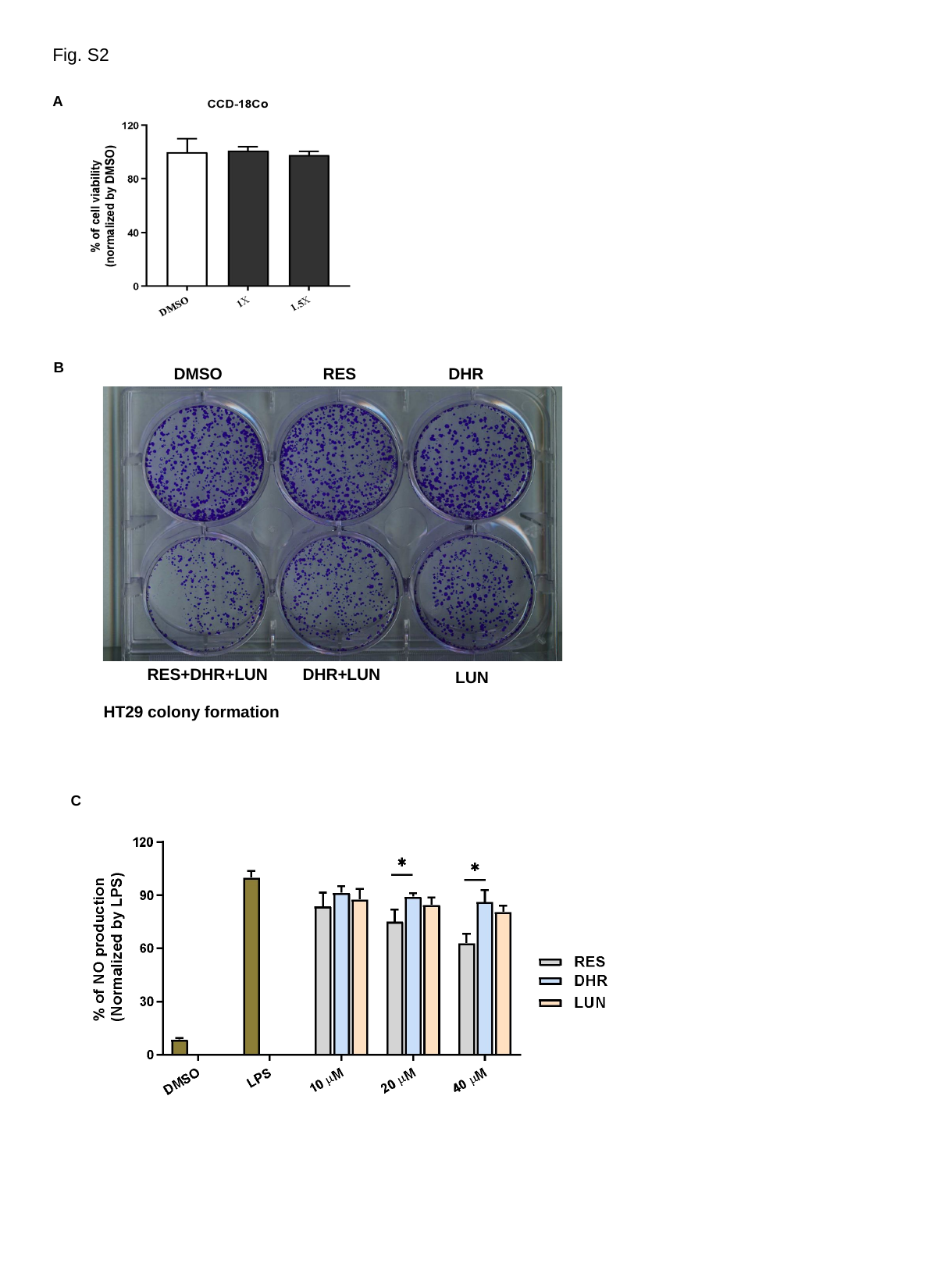

Fig. S2
A
B
RES
DHR
DMSO
RES+DHR+LUN
DHR+LUN
LUN
HT29 colony formation
C
